# Efficient chemical-disease identification and relationship extraction using Wikipedia to improve recall

**DOI:** 10.1093/database/baw039

**Published:** 2016-04-08

**Authors:** Daniel M. Lowe, Noel M. O’Boyle, Roger A. Sayle

**Affiliations:** NextMove Software Ltd, Innovation Centre, Unit 23, Science Park, Milton Road, Cambridge, United Kingdom

## Abstract

Awareness of the adverse effects of chemicals is important in biomedical research and healthcare. Text mining can allow timely and low-cost extraction of this knowledge from the biomedical literature. We extended our text mining solution, LeadMine, to identify diseases and chemical-induced disease relationships (CIDs). LeadMine is a dictionary/grammar-based entity recognizer and was used to recognize and normalize both chemicals and diseases to Medical Subject Headings (MeSH) IDs. The disease lexicon was obtained from three sources: MeSH, the Disease Ontology and Wikipedia. The Wikipedia dictionary was derived from pages with a disease/symptom box, or those where the page title appeared in the lexicon. Composite entities (e.g. heart and lung disease) were detected and mapped to their composite MeSH IDs. For CIDs, we developed a simple pattern-based system to find relationships within the same sentence. Our system was evaluated in the BioCreative V Chemical–Disease Relation task and achieved very good results for both disease concept ID recognition (F_1_-score: 86.12%) and CIDs (F_1_-score: 52.20%) on the test set. As our system was over an order of magnitude faster than other solutions evaluated on the task, we were able to apply the same system to the entirety of MEDLINE allowing us to extract a collection of over 250 000 distinct CIDs.

## Introduction

Identifying the relationships between chemicals and diseases has many applications in biomedical research and healthcare. The BioCreative V CDR (Chemical–Disease Relation) task was organized to encourage research into text mining in this area and to evaluate current solutions. The challenge was formed of two subtasks; the first was to identify diseases and normalize them to MeSH (Medical Subject Headings) IDs. The second was to identify causal relationships between chemicals and diseases, with the results reported as MeSH ID pairs.

The corpus was formed of 1500 MEDLINE articles divided into 3 sets of 500. The training and development sets were provided to participants, whereas the test set was provided only after the system evaluation had been performed. All articles were formed of the title and abstract and had been manually annotated by curators from the Comparative Toxicogenomics Database (1). These annotations included chemicals, diseases and CDRs. Where possible, the corresponding MeSH ID was also given for each concept. These IDs were used when evaluating solutions and hence required participating systems not just to identify chemical/disease terms but to know which concept they corresponded to. The test set was evaluated by participants providing a web service which identified diseases and/or CDRs. The articles in the test set were provided as input to these web services and the output used to evaluate participants’ solutions for precision/recall and responsiveness. Further information about the challenge is available in the challenge task articles (2, 3).

## Disease recognition and mapping to MeSH

To facilitate mapping of entities to MeSH IDs, we used a dictionary-based approach. The dictionary was derived from three sources: MeSH (4), the Disease Ontology (5) and Wikipedia (6).

### MeSH

The US National Library of Medicine provided ‘MeSH descriptors and qualifiers’ and the ‘Supplementary Concept Records‘files. From these, all terms (and synonyms thereof) which were in MeSH trees C (Disease) or F03 (Mental Disorders) were extracted together with their MeSH ID mapping. The following tree branches were excluded: C23.550.291 (Disease attributes), C23.550.260 (Death) and C26 (Wounds and Injuries (unspecified)). These branches were excluded as, by some definitions, they are not diseases. MeSH supplementary records were selected if they referred to an included disease MeSH ID.

### Disease ontology

Concept titles and their synonyms were extracted from the Disease Ontology. Where a cross-reference to MeSH was present, these terms were associated with the corresponding MeSH ID.

### Wikipedia

**Figure 1.**
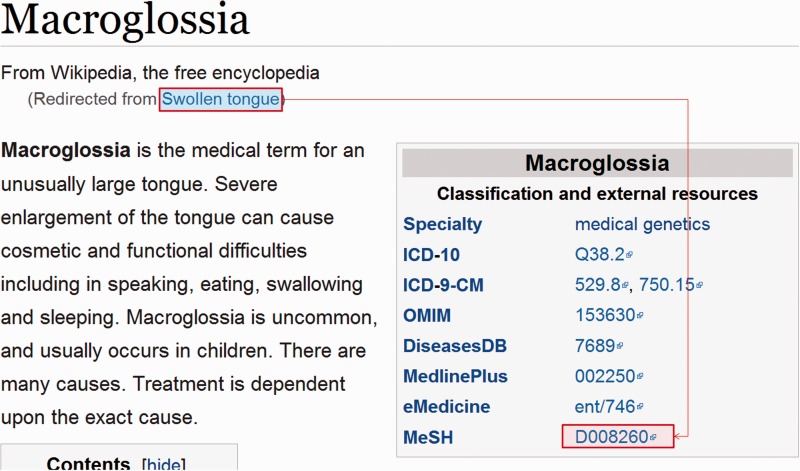
Example of a Wikipedia disease page, demonstrating the term relationships that were extracted in bulk from a dump of Wikipedia.

A dump of current Wikipedia page articles (enwiki-20150602-pages-articles.xml.bz2) was downloaded. Pages with disease or symptom boxes, that contained a link-out to MeSH, were identified. From these, the page title and all redirects to the page were recorded as mapping to that MeSH ID (Figure 1). Occasionally, a MeSH tree number was used instead of an ID, requiring these to be converted to the corresponding ID. A large collection of redirects to be ignored was empirically assembled e.g. ‘Allergy medication’, ‘HPV test’, ‘History of acne’, ‘Rehydrated’, ‘2009 flu pandemic’. These highlight the problem that, while Wikipedia pages are a very rich source of synonyms (especially common names and adjectival forms), the redirects are not semantic. A small collection of page titles to be ignored was also assembled for those pages that did not actually relate to a disease, e.g. MUMPS (a programming language).

Additionally, if a page title in Wikipedia matched a term in the dictionary assembled from MeSH and the Disease Ontology, it was assumed to relate to the same concept and, hence, all redirects to the page were linked to the MeSH ID used by the term in the dictionary.

### Final dictionary preparation

A final dictionary was assembled by adding the source dictionaries in the following order: manually curated dictionary (mostly used to correct MeSH IDs referenced from Wikipedia terms), MeSH terms, Disease Ontology terms, terms taken from the training/development corpus and terms taken from Wikipedia. Spelling variants, e.g. tumor vs tumour, were generated at the point of adding a term to the dictionary. If a term appeared in two source dictionaries, the first dictionary added determined the MeSH ID used in the final dictionary.

A stop word list was used to exclude terms that were unwanted, either due to not being a disease name (e.g. ‘birth weight’) or frequently having a non-disease related meaning (e.g. ‘sterile’). Some of the most common types of term that required inclusion in the stop word list were gene names and short abbreviations. We also added disease names which were followed by their corresponding abbreviation in brackets, as these should be recognized as two entities.

Index names, such as ‘Abnormality, Congenital’, were uninverted by splitting on comma space and rearranging. Cases where a list was intended (e.g. ‘, or’) were left unchanged. Qualifiers (e.g. ‘, with’) were moved to the end of the term with the comma removed. Finally, modified versions of existing terms, which could be expected to be a synonym or refinement of the original term, were generated and added to the dictionary e.g. ‘infection’ replaced by ‘disease’, ‘cancer’ replaced by ‘carcinoma’.

The development of the scripts to extract the terms from Wikipedia, the Disease Ontology and MeSH, as well as the manual preparation of the stop word list took ∼2 weeks.

### Disease recognition with LeadMine

LeadMine (7) was configured to use this dictionary for recognition and for normalizing recognized entities to MeSH IDs. A low level of spelling correction was used to recognize and correct minor spelling errors. After recognition, composite entities, e.g. ‘heart and lung disease’, were detected and mapped to MeSH IDs, corresponding to the reconstructed entities i.e. ‘heart disease’ and ‘lung disease’. This worked as follows:

Look for ‘and’ or ‘or’ preceding a multi-word recognized entity

Take the word before the ‘and’ or ‘or’ together with the last word of the recognized entity to construct a potential entity

If this potential entity would be recognized by the dictionary, then add the potential entity.

As a special case, where MeSH distinguished the drug-induced form of a disease, the MeSH ID for the drug-induced form was always chosen.

### Effect of adding Wikipedia dictionary

By adjusting the source dictionaries, the performance change of including the Wikipedia terms was quantified on the development set (Table 1). Our final system corrected some of the mistakes in the Wikipedia terms e.g. heart disease linked to the MeSH ID for cardiovascular disease, as heart disease is not currently a distinct page on Wikipedia but instead redirects to cardiovascular disease.

**Table 1.** Effect of the choice of lexicon on performance of the system on the development set.

**Dictionaries**	**Precision**	**Recall**	**F_1_-score**
Wikipedia	79.3%	61.3%	69.1%
MeSH/Disease Ontology	91.6%	67.1%	77.4%
MeSH/Disease Ontology/Wikipedia	85.1%	73.1%	78.6%

## Chemical-induced disease relationship extraction

### Chemical recognition with LeadMine

LeadMine was used to detect chemical entities, using a configuration similar to that used in the CHEMDNER-Patents task (8) but with the addition of a dictionary of special cases noted in the annotation guidelines, e.g. oral contraceptive. The terms in the chemical branch of MeSH were used to resolve recognized terms to MeSH IDs. Where an exact match was not found variants were tried, e.g. plural of recognized term. This achieved an F_1_-score of 92.3% for chemical MeSH ID recognition on the development set.

### Relationship detection

**Figure 2.**
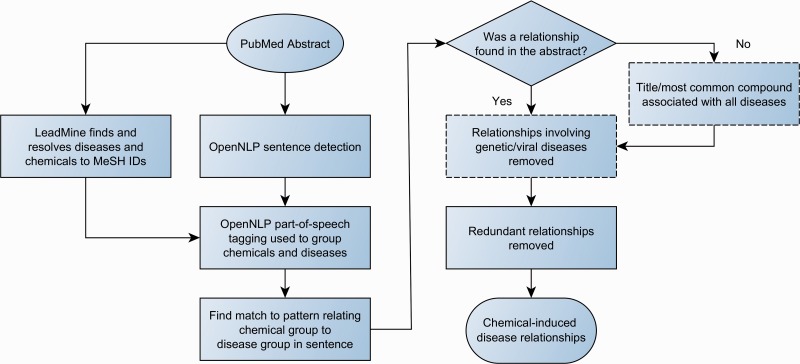
Workflow for chemical-disease relationship extraction. Dashed boxes are optional steps.

The workflow for relationship detection is summarized in Figure 2.

Sentence detection and part of speech tagging were performed by OpenNLP (9). Using a method similar to Schlaf *et al.* (10), the part of speech tags were used to group diseases/chemicals by grouping all entities that were not separated by a verb, preposition or sub-ordinating conjunction. Patterns were used to identify relationships between chemical and disease groups. Most patterns were regex-based, typically consisting of attempting to find a key word/phrase. For example, chemical <caused> disease, where caused is:

.*(-associated|(?<!not |[a-z])(associated with|cause[sd]|…))(?! no) .*

As can be seen from the pattern, a simple attempt is made to avoid identifying negative associations. Tables 2 and 3 summarize the patterns used and their precision, when evaluated on the union of the training and development sets.

**Table 2.** Precision of patterns where the chemical term precedes the disease term.

**Pattern**	**True Positives**	**False Positives**	**Precision**
Chemical <caused>	528	219	70.7%
Chemical Disease	41	25	62.1%
Chemical <related to >	8	2	80.0%
<negative effects caused by> chemical	4	2	66.7%
<relationship between> chemical <and>	2	1	66.7%

**Table 3.** Precision of patterns where the chemical term follows the disease term.

**Pattern**	**True Positives**	**False Positives**	**Precision**
Disease <caused by >	208	79	72.47%
Disease <after or during>	108	76	58.70%
Disease <after or while taking>	73	36	67.00%
Disease <in person taking>	18	4	81.80%
Disease <effect of >	14	14	50.00%
Disease <related to >	14	6	70.00%
Disease <complications of >	12	5	70.60%
<induction of> Disease <by or with>	2	1	66.70%

Patterns were developed by taking a sentence containing a chemical and disease known to be in a chemical-induced disease relationship (CID), and manually identifying the key word/phrase that indicated the relationship. This gives the prototypical relationship pattern which is then expanded by identifying and postulating other synonymous phrases. Precision was evaluated using the relationships that had been provided for the training and development sets. As the curators only included the most specific relationships, a relationship that in isolation would be considered correct, could nonetheless be counted as a false positive if the document contained a more specific relationship. As a result, many of the ‘false positives’ would not have been actually been considered to be incorrect when the patterns were developed on a per sentence basis.

All diseases/chemicals in a group linked by one of these patterns were identified as being in CIDs. When no patterns matched an abstract, optionally, a heuristic is applied to find likely relationships. All chemicals in the title, or failing that the first most commonly mentioned chemical in the abstract, are associated with all diseases in the entire abstract. As the patterns only found relationships within a sentence, this heuristic was the only mechanism through which relationships between entities in different sentences were found.

Optionally a filtering step was performed. A small number of diseases were blocked: D064420, D010300, D003643, D066126 and D020258, as they were too vague. Additionally a disease’s corresponding MeSH tree numbers were used to block C02 (Virus Diseases) and C16.320 (Genetic Diseases, Inborn) as these are unlikely to be caused by chemicals.

In all cases, MeSH tree numbers were used to identify redundant relationships, i.e. those in which the tree numbers of a disease are entirely refinements of those used in another relationship.

The patterns, and framework to identify entities in relationships indicated by them, were developed over a period of 2 weeks.

## Evaluation

Tomcat was installed on an Amazon Web Services instance with 2 GiB of RAM and 1 core. Disease named entity recognition (DNER) was evaluated on the agreement between the system’s MeSH IDs and those in the test set. For CIDs, our three runs correspond to the pattern-based system, that system plus filters to improve precision, and the previous system plus the heuristic to find the most likely CDR. Our results, as measured by the task organizers on the test set, are summarized in Table 4.

**Table 4.** Performance of the system on the test set for the DNER and CID tasks

**Task**	**Precision**	**Recall**	**F_1_-score**	**Response time**
DNER	86.08%	86.17%	86.12%	45.0 ms
CID (pattern-based)	57.65%	36.77%	44.90%	96.9 ms
CID (pattern-based with filters)	60.99%	35.93%	45.22%	121.8 ms
CID (pattern-based with filters and recall increasing heuristic)	52.62%	51.78%	52.20%	119.3 ms

Our system performed well on the DNER task, with an F_1_-score approaching the inter-annotator agreement of 88.75% (3). When compared with other participants our system had very high recall, with the one system that had higher F_1_-score achieving this through significantly higher precision at significantly lower recall (11). Both our system’s high recall and relatively low precision can be attributed to our use of Wikipedia. When our dictionary of diseases was built without the use of Wikipedia, our DNER results on the test set were 90.49% precision, 80.94% recall and 85.45% F_1_-score, i.e. the use of Wikipedia improved recall by 5%. As the Wikipedia dictionary contains many terms that are entirely absent from the MeSH hierarchy, it is likely that these terms were more often missed or misannotated by the curators.

Our system’s results for CID identification compared favorably with other participants, with only the two systems that employed databases of known CDRs performing better (12, 13). Using the now available test set, we evaluated what performance could be achieved if the gold-standard entities were used i.e. the DNER step was ‘perfect’ (Table 5). As would be expected, this improved the results significantly. However, the results are still low in the absolute sense, highlighting the difficulty of the relationship extraction part of this task.

**Table 5.** Performance of the system on the test set for CID identification when using gold-standard entities.

**Task**	**Precision**	**Recall**	**F_1_-score**
CID (pattern-based, gold-standard entities)	62.75% (+5.10%)	44.56% (+7.79%)	52.11% (+7.21%)
CID (pattern-based with filters, gold-standard entities)	66.52% (+5.53%)	43.62% (+7.69%)	52.69% (+7.47%)
CID (pattern-based with filters and recall increasing heuristic, gold-standard entities)	59.29% (+6.67%)	62.29% (+10.51%)	60.75% (+8.55%)

Change in performance from using gold-standard entities in parenthesis.

Due to LeadMine’s speed of annotation, the response time for DNER is likely to be primarily limited by internet latency. To simplify implementation, the DNER configuration performed both chemical and disease recognition.

## Application to MEDLINE

MEDLINE (14) is a database containing over 22 million journal references. These records include bibliographic data, the title, and, in many cases, the abstract. We downloaded the current version of the MEDLINE database as of 28 September 2015. As the majority of documents are not expected to contain a CID relationship, the assumption made by the recall boosting heuristic that tries to find the chemical most likely to be in such a relationship is invalid. Hence, we used the ‘CID (pattern-based with filters)’ configuration to maximize precision.

Relationships were looked for in both the title and abstract text of each record, or, where the abstract text was unavailable, just in the title text. Processing took <6 h on a desktop PC (Core i7-2600) using four threads. The output consisted of a tab separated file containing:

- Record PubMedId
- Chemical MeSH ID
- Disease MeSH ID
- chemical name found in the text
- disease name found in the text
- Passage of text in which the relationship was found.

E.g.

**Table:** 

23427516	C052342	D007007	topiramate	Hypohidrosis	Hypohidrosis and hyperthermia during topiramate treatment in children.
23427516	C052342	D005334	topiramate	hyperthermia	

The resulting set contained 282 604 distinct CID relationships, with 860 640 records containing at least one relationship. As would be expected, the majority of records did not contain a relationship. The inclusion of the passage of text allows in many cases for the relevancy of the relationship to be quickly accessed.

The most commonly associated relationship was Streptozotocin with diabetes Mellitus, in which it is used to induce diabetes in animal models.

Manual inspection of the results highlighted numerous ways in which the precision of the system could be improved. A common error was that two disease terms were found within ‘tumor necrosis factor’ (a protein family). This can be resolved by also identifying protein names. Another common error was in cases where a chemical was associated with a disease but the relationship was an ameliorative one. As the challenge’s corpus contained relatively few examples of such relationships, the patterns are currently less robust to identifying cases that invert the meaning of the phrase e.g. ‘Ritodrine therapy associated with “remission of” pemphigoid gestationis’. The current system is robust to some such patterns, e.g. ‘disease improved after taking chemical’ is not recognized as a CID.

## Conclusions

Our approach to disease recognition allows rapid identification of diseases with excellent recall and precision. The use of Wikipedia significantly improved the system’s ability to recognize adjectival and trivial names for diseases. Our simple pattern-based approach to CDR extraction proved to be relatively precise, as well as fast, allowing its trivial application to a collection of texts many orders of magnitude larger than that used in the BioCreative CDR task. The CIDs, obtained by processing the entirety of MEDLINE (282 604 relationships), could be immediately useful as a resource for looking up these relationships, or as an aid to curators manually populating databases of these relationships.

*Conflict of interest*. The authors are employees of NextMove Software Ltd. who develop and license the text-mining solution described in this paper.
